# Mosaic Evolution of Brainstem Motor Nuclei in Catarrhine Primates

**DOI:** 10.1155/2011/236894

**Published:** 2011-05-29

**Authors:** Seth D. Dobson, Chet C. Sherwood

**Affiliations:** ^1^Department of Anthropology, Dartmouth College, Hanover, NH 03755, USA; ^2^Department of Anthropology, The George Washington University, Washington, DC 20052, USA

## Abstract

Facial motor nucleus volume coevolves with both social group size and primary visual cortex volume in catarrhine primates as part of a specialized neuroethological system for communication using facial expressions. Here, we examine whether facial nucleus volume also coevolves with functionally unrelated brainstem motor nuclei (trigeminal motor and hypoglossal) due to developmental constraints. Using phylogenetically informed multiple regression analyses of previously published brain component data, we demonstrate that facial nucleus volume is not correlated with the volume of other motor nuclei after controlling for medulla volume. Our results show that brainstem motor nuclei can evolve independently of other developmentally linked structures in association with specific behavioral ecological conditions. This finding provides additional support for the mosaic view of brain evolution.

## 1. Introduction

Two competing models of brain evolution have dominated the neuroscience literature over the past 15 years. The first posits that the interspecific scaling of vertebrate brain components is explained mostly by a conserved pattern of neurogenesis, such that structures that develop later tend to be relatively large [1, 2]. This is supported by the fact that later developing structures exhibit larger allometric exponents when scaled against overall brain size [1]. Supporters of the developmental correlation model argue that brain structure evolves due primarily to selection on overall brain size, as opposed to the specialization of particular areas for specific functions [2]. Thus, individual brain structures vary in size according to general scaling principles that constrain adaptive evolution, thereby limiting the impact of behavioral ecological conditions on brain structure.

The alternative model posits that natural selection can act to expand or contract the size of individual brain components, independent of overall brain size, without necessarily altering the size of functionally unrelated regions [3, 4]. Supporters of this mosaic evolution model argue that the coordinated evolution of individual brain regions is due to functional and/or structural connections [5, 6]. According to this model, developmental constraints can be overridden by selection to enlarge separate neural systems in response to specific behavioral ecological conditions. This idea is supported by comparative analyses of neural specialization in species as diverse as primates [7], birds [8], and fish [9]. The mosaic evolution model also posits a role for constraints, but supporters of this model tend to emphasize energetic trade-offs influencing overall brain size [10] rather than developmental correlations per se [11].

In a previous paper, we examined the coordinated evolution of brain regions involved in producing and processing facial expressions in anthropoid primates [12]. The results of our study revealed that social group size is positively correlated with the relative size of the facial motor nucleus, which sends motor neurons from the brainstem to the muscles of facial expression [13]. This pattern, which we observed in catarrhines but not platyrrhines, is consistent with the idea that facial communication is an important form of conflict management and bonding within catarrhine social groups [14–16]. In addition, we found that facial nucleus volume is positively correlated with primary visual cortex volume, after controlling for the size of the rest of the brain [12]. These results bolster the mosaic view of brain evolution. However, they do not preclude the possibility of developmental correlations within the brainstem, that is, correlated size changes in functionally unrelated motor nuclei.

The purpose of the present study is to test the hypothesis that the size of the facial motor nucleus in catarrhines evolves in coordination with other brainstem motor nuclei due to developmental correlation. Developmental covariation among brainstem motor nuclei is to be expected since these nuclei show similar patterns of growth-factor receptor expression and coordinated modulation of neuronal proliferation and survival [17]. We will examine two comparative predictions of the developmental correlation model: (i) facial nucleus size is positively correlated with trigeminal motor nucleus size and hypoglossal nucleus size, after controlling for the size of the medulla and (ii) the relative sizes of the trigeminal motor nucleus and hypoglossal nucleus are positively correlated with social group size. The former prediction addresses the essence of the developmental correlation model: coordinated size changes due to a shared developmental basis. The latter prediction derives from the fact that facial nucleus size is correlated with group size [12]. To assess the specificity of this group-size effect, we examine the possibility that the other two brainstem orofacial motor nuclei are also correlated with group size. The results of our study contribute to debates regarding the relative importance of developmental constraints versus adaptive specializations in mammalian brain evolution.

## 2. Materials and Methods

Brain component volumes for 14 group-living, nonhuman catarrhine species were taken from previously published sources [13, 18, 19]. Group size data were taken from an unpublished dataset available on C. Nunn's website [20]  (http://www.people.fas.harvard.edu/~nunn/index.html). We examined trait correlations using multiple regression analyses. Two sets of analyses were carried out: (i) we examined the volume of the trigeminal motor nucleus and hypoglossal nucleus in relation to facial nucleus volume after controlling for medulla size [13] and (ii) we examined the relative volume of the trigeminal motor nucleus and hypoglossal nucleus in relation to group size. Autocorrelation, which can occur when the independent variable represents a large part of the dependent variable, is not a serious issue for our analyses because each nucleus comprises less than 0.5% of the volume of the total medulla [21]. All data were log-transformed (natural) prior to analysis. 

Regression coefficients and standard errors were generated using a phylogenetic generalized least-squares (PGLSs) approach [22]. We used COMPARE 4.6b [23] to perform PGLSs multiple regressions based on a single consensus tree with chronometric branch lengths [24], which we downloaded from the 10kTrees website (version 2) (http://10ktrees.fas.harvard.edu/). To take into account phylogenetic uncertainty, we also ran each analysis on a block of trees (*N* = 1000) in which the position of any given node varies as a function of its Bayesian posterior probability [24]. The null hypothesis (slope = 0) was assessed using 95% confidence intervals for each regression coefficient [25]. In the case of the tree block analyses, we incorporated both sampling variance and variance due to phylogenetic uncertainty into the calculation of confidence intervals [26].

## 3. Results and Discussion

**Figure 1:**
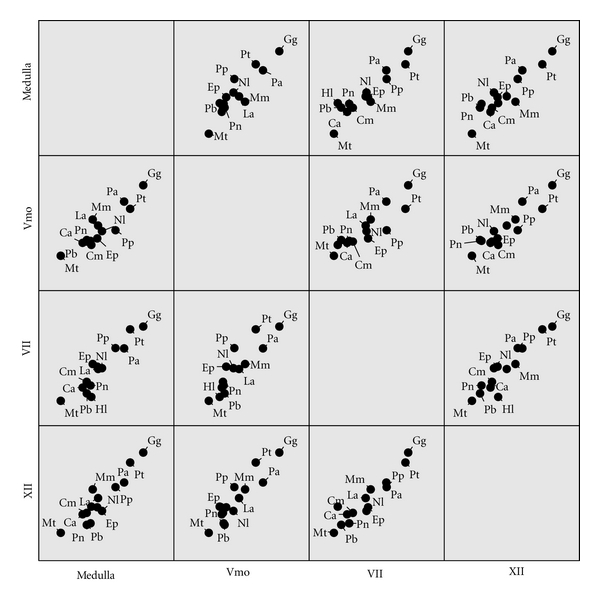
Scatter-plot matrix depicting relationships among brainstem motor nuclei volumes in catarrhines before size correction. Data are natural log transformed. Variable abbreviations—medulla: medulla volume; Vmo: trigeminal motor nucleus volume; VII: facial motor nucleus volume; XII: hypoglossal nucleus volume. Species abbreviations—Ca: *Cercopithecus ascanius*; Cm: *Cercopithecus mitis*; Ep: *Erythrocebus patas*; Gg: *Gorilla gorilla*; Hl: *Hylobates lar*; La: *Lophocebus albigena*; Mm: *Macaca mulatta*; Mt: *Miopithecus talapoin*; Nl: *Nasalis larvatus*; Pp: *Pan paniscus*; Pt: *Pan troglodytes*; Pa: *Papio anubis*; Pb: *Procolobus badius*; Pn: *Pygathrix nemaeus*.

Figure 1 demonstrates the strong degree of covariation between brainstem orofacial motor nuclei in catarrhines prior to size correction. However, the multiple regression results in Table 1 indicate that this pattern of covariation disappears after controlling for medulla size, that is, neither trigeminal motor nucleus volume nor hypoglossal nucleus volume is a significant predictor of facial nucleus volume independent of medulla volume. Moreover, social group size is not positively correlated with either trigeminal motor nucleus volume or hypoglossal nucleus volume after size correction (Table 1). The results of the tree block analyses are identical with the consensus tree results because the degree of phylogenetic uncertainty in our sample is negligible. Thus, the hypothesis that catarrhine brainstem motor nuclei evolve in coordination with each other due to a shared developmental basis is not supported by our results. Instead, it appears that relative facial motor nucleus size evolves independently of the rest of the medulla and in association with social group size.

Taken together, the results of our previous work [12] and the present study provide additional support for the mosaic model of brain evolution [3]. Proponents of this model assert that natural selection can target functionally interconnected neural systems resulting in structural changes that are relatively unconstrained by developmental processes [11]. We found that the catarrhine facial motor nucleus evolves independently of other brainstem orofacial motor nuclei in response to a specific behavioral ecological condition, group size, and in coordination with a functionally linked region, the primary visual cortex [12]. The latter result is particularly striking because it involves coevolution between two brain components that are not structurally interconnected by direct axonal pathways. Moreover, because the medulla and neocortex undergo neurogenesis at different times [1], developmental correlation is an unlikely explanation for this pattern of correlated evolution. 

It appears that general trends in brain evolution observed at higher taxonomic levels can mask adaptive diversity at lower taxonomic levels. For example, the negative relationship between relative neocortex size and relative limbic structure size observed at the ordinal level in mammals does not apply within orders [27]. Similarly, it has been suggested that the mammalian central visual system exhibits a high degree of evolutionary conservatism [28]. However, numerous studies of the primary visual cortex in primates have demonstrated that species can deviate from allometric trends as a function of activity period, dietary preference, and/or sociality [7, 12, 29–32]. Thus, it seems that much of the debate concerning the relative importance of adaptive specialization versus developmental constraint is driven by differences in the taxonomic sampling.

## 4. Conclusions

Previous research has shown that correlated evolution may occur within structurally interconnected neural systems [5, 6]. Our findings are unique in demonstrating that mosaic brain evolution can also involve coordinated changes in the volume of brain components that are not structurally linked by direct axonal pathways, but that participate in a common adaptive complex [12]. These results also provide further support for the idea that neural specializations in mammals are not restricted to executive brain functions. Brainstem structures can also undergo adaptive specialization in response to the motor and/or sensory demands of specific behavioral ecological conditions [33–37].

## Figures and Tables

**Table 1:** Multiple regression results using phylogenetic generalized least squares. Results are presented for a single consensus tree. The results of the tree block analyses are identical to the consensus tree results because the degree of phylogenetic uncertainty in our sample is negligible (see Materials and Methods for further details). CI: confidence interval (±2 standard errors); Vmo: trigeminal motor nucleus volume; VII: facial motor nucleus volume; XII: hypoglossal nucleus volume.

Dependent	*R* ^2^	Independent	Slope	95% CI
VII	91%	Medulla	0.61	0.03, 1.19
		Vmo	−0.08	−0.78, 0.62
		XII	0.53	−0.07, 1.13
Vmo	86%	Medulla	0.89	0.67, 1.11
		Group size	0.09	−0.05, 0.23
XII	86%	Medulla	1.04	0.78, 1.30
		Group size	0.08	−0.08, 0.24
